# Achondroplasia: Current Disease-Modifying Therapies and Genome Editing Strategies

**DOI:** 10.3390/ijms27198571

**Published:** 2026-09-25

**Authors:** Ekaterina Kondrateva, Svetlana Smirnikhina

**Affiliations:** Research Centre for Medical Genetics, Moscow 115522, Russia

**Keywords:** achondroplasia, FGFR3-selective inhibition, disease-modifying therapy, gene therapy, genome editing, enhancer targeting, gene regulation, delivery to cartilage, rare genetic disease

## Abstract

Achondroplasia (ACH) is the most common non-lethal skeletal dysplasia and is caused in the vast majority of cases by a heterozygous gain-of-function variant in fibroblast growth factor receptor 3 (FGFR3, p.Gly380Arg). Hyperactive FGFR3 signaling constrains endochondral bone growth by suppressing growth-plate chondrocyte proliferation and hypertrophic differentiation. In the past decade, management has expanded beyond supportive care, and several mechanism-based interventions have shown disease-modifying effects in clinical studies. These include the activation of the NPR2/CNP axis with vosoritide and navepegritide and the pharmacologic attenuation of FGFR signaling with infigratinib, while extracellular biologics designed to modulate ligand–receptor interactions remain investigational. In parallel, gene-based approaches are increasingly discussed as routes toward the etiology-directed control of the causal driver, ranging from the cartilage-biased tuning of FGFR3 expression to variant-level genome editing concepts. Here, we synthesize the current therapeutic landscape, explicitly distinguish pathway-targeted disease modification from etiology-directed genetic strategies, and highlight the key translational barriers for gene-based approaches, including target-tissue delivery to the growth-plate, dose control and durability across development, redosing and immunogenicity constraints, and long-term safety.

## 1. Introduction

Achondroplasia (ACH; OMIM #100800) is an autosomal dominant skeletal dysplasia characterized by disproportionate short stature with rhizomelic limb shortening, macrocephaly with frontal bossing, and midface hypoplasia, with clinically important complications, including foramen magnum stenosis, sleep-disordered breathing, recurrent otitis media/hearing loss, spinal stenosis, and orthopedic problems [1]. The worldwide birth prevalence of achondroplasia has been estimated at approximately 4.6 per 100,000 births [2], making it the most common form of genetic short-limbed dwarfism [3]. Most cases arise de novo and show a paternal age effect [4]. At the molecular level, achondroplasia is overwhelmingly caused by FGFR3 p.Gly380Arg, most commonly resulting from c.1138G>A and in approximately 1% from c.1138G>C [1,4,5]. Because the disorder is driven by a single hyperactive signaling node rather than a missing enzyme or structural protein, classical replacement paradigms are not directly applicable.

For decades, the management of achondroplasia has been largely conservative and complication-oriented, aiming to mitigate orthopedic, neurologic, and respiratory sequelae rather than to directly neutralize the causal FGFR3 gain-of-function itself. Surgical limb lengthening remains a non-molecular option for selected patients with achondroplasia, although it involves substantial treatment burden and a risk of serious complications [4]. While outside the main scope of this review, it provides relevant clinical context for evaluating the magnitude and goals of pharmacological growth-promoting therapies. Advances in growth plate signaling biology have enabled a shift toward pathway-targeted disease modification and, potentially, etiology-directed genetic strategies. In this review, we synthesize the current evidence across therapeutic modalities and critically assess mechanistic rationale, maturity of data, and translational constraints; throughout, we use “etiology-directed” to denote interventions that directly correct, selectively suppress, or durably transcriptionally down-tune the causal FGFR3 gain-of-function at the DNA/RNA/cis-regulatory level, in contrast to pharmacological pathway modulators. The current therapeutic landscape for achondroplasia is summarized in Figure 1.

Throughout the review, clinical efficacy is distinguished from preclinical or conceptual feasibility, and genetic approaches that have not been evaluated in patients with achondroplasia are discussed as experimental strategies rather than established therapies.

## 2. Literature Search Strategy

This article was prepared as a narrative review. The relevant literature was identified primarily through PubMed/MEDLINE using combinations of terms related to “achondroplasia”, “FGFR3”, disease-modifying therapies, individual therapeutic agents, genome editing, gene regulation, growth plate, and cartilage delivery. Clinical development and regulatory status were additionally verified using ClinicalTrials.gov, the Japan Registry of Clinical Trials (jRCT), and publicly available documents from the U.S. Food and Drug Administration (FDA) and European Medicines Agency (EMA). The literature and trial-status search was updated through 15 September 2026. Peer-reviewed original studies were prioritized for efficacy, safety, and mechanistic conclusions; relevant reviews were used for background information, while regulatory documents and trial registries were used primarily to establish approval and development status when peer-reviewed information was unavailable. This review is intended as a narrative synthesis and did not follow a formal systematic-review or meta-analysis protocol.

## 3. Pathophysiology and Targetable Nodes

FGFR3 is a negative regulator of endochondral bone growth. In the growth plate, FGFR3 signaling limits chondrocyte proliferation and delays hypertrophic differentiation, thereby restraining longitudinal bone growth [6]. The achondroplasia-associated p.Gly380Arg substitution increases FGFR3 signaling and shifts growth-plate dynamics toward reduced proliferative zone expansion and impaired hypertrophic maturation [4]. Among downstream effectors, the RAS-RAF-MEK-ERK mitogen-activated protein kinase (MAPK) cascade is a central mediator and a recurrent pharmacological target [6].

A therapeutically important endogenous counter-regulatory axis is mediated by C-type natriuretic peptide (CNP) signaling through natriuretic peptide receptor B (NPR-B/NPR2), which functionally antagonizes FGFR3 signaling in growth plate chondrocytes and supports endochondral growth [7]. This biology underpins the clinical development of CNP analogs and long-acting CNP prodrugs. In parallel, small-molecule kinase inhibitors target FGFR signaling more directly, while extracellular biologics seek to modulate ligand–receptor interactions and the local ligand milieu around cartilage.

From a therapeutic point of view, this pathway organization is important because it creates several levels at which the pathological signal can be reduced without necessarily correcting the DNA variant itself. The most distal level is the functional antagonism of MAPK output by CNP/NPR2 signaling. A more proximal level is the pharmacological inhibition of the FGFR kinase domain, which directly reduces receptor signaling but may also affect FGFR1 and FGFR2, depending on selectivity. The most proximal level is the genetic control of the FGFR3 driver, by correcting the pathogenic allele, selectively suppressing the mutant transcript, or reducing receptor expression in growth-plate cartilage. These levels differ in expected durability, reversibility, and safety requirements, and therefore should not be considered interchangeable therapeutic classes [6,7].

The same biology explains why the complete suppression of FGFR3 is not the desired objective. FGFR3 acts as a physiological brake on endochondral growth; achondroplasia reflects excessive braking rather than the absence of a growth-promoting factor. Therefore, the most plausible molecular goal is not maximal pathway inhibition but the restoration of an acceptable signaling range. This is especially relevant for pediatric treatment, where the intervention may need to operate for many years during active growth and where both under-treatment and over-suppression could have long-term skeletal consequences [6].

## 4. First-Generation Pharmacological Pathway Modulators

### 4.1. CNP Analogs and NPR2 Pathway Amplification

Vosoritide (Voxzogo™; BMN 111) is the first approved disease-modifying therapy for ACH and is administered once daily by subcutaneous injection. It is a modified CNP analog engineered to be resistant to proteolytic degradation by neutral endopeptidase (NEP). By stimulating NPR2 and increasing cGMP signaling, vosoritide counterbalances FGFR3-mediated MAPK activation in growth-plate chondrocytes [7]. In a randomized, double-blind, placebo-controlled phase 3 trial in children with achondroplasia, vosoritide increased annualized growth velocity compared with placebo over 52 weeks, with an adjusted mean difference of 1.57 cm/year (95% CI, 1.22 to 1.93; *p* < 0.0001) [8]. In the open-label phase 3 extension, continued treatment was associated with persistent growth-promoting effects over 2 years [9]. Vosoritide is authorized for use in pediatric patients with achondroplasia and open epiphyses [10,11].

Longer follow-up is especially important because CNP-based therapies are intended for use across several years of active growth. In a 2025 analysis of the ongoing phase 3 extension study, 119 children treated with vosoritide were followed for up to 6 years. The study reported sustained growth-promoting effects, a favorable safety profile over 464.05 person-years of exposure, and no new long-term harms or deaths; three-year comparisons also suggested additional height gain and improvement in upper-to-lower body segment ratio in younger children [12]. These data strengthen the clinical position of vosoritide while also emphasizing the need for long-term endpoints beyond annualized growth velocity. However, safety beyond the currently available follow-up and through skeletal maturity remains to be established.

A practical limitation of native CNP is its very short plasma half-life (2–3 min), motivating the development of more stable and long-acting CNP analogs and prodrugs. Navepegritide (TransCon CNP) is a long-acting prodrug engineered for once-weekly subcutaneous dosing that releases active CNP over time. In the randomized, placebo-controlled phase 2b APPROACH trial (NCT05598320; *n* = 84 children aged 2–11 years), weekly navepegritide (100 μg/kg/wk) was superior to placebo for annualized growth velocity at week 52, with least-squares mean AGV 5.89 cm/y vs. 4.41 cm/y and an LS mean difference of 1.49 cm/y (95% CI, 1.05 to 1.93; *p* < 0.001) [13]. The trial also reported improvements in additional exploratory outcomes related to lower-limb alignment and patient-reported physical functioning, alongside a generally similar tolerability profile versus placebo and an ongoing open-label extension [13]. Long-acting CNP approaches may therefore address adherence and injection burden while maintaining the biological rationale of NPR2 pathway amplification. In February 2026, the FDA granted accelerated approval to Yuviwel (navepegritide) to increase linear growth in pediatric patients 2 years of age and older with achondroplasia with open epiphyses; continued approval may be contingent upon the verification and description of clinical benefit in confirmatory trials [14]. Another long-acting CNP-based approach, BMN 333, is currently being evaluated in the phase 2/3 ASPEN study (NCT07441876) in children with achondroplasia [15].

Taken together, the CNP-based therapies provide the strongest clinical proof that the molecular modulation of growth-plate signaling can change the natural history of linear growth in achondroplasia. However, they also illustrate the limits of pathway compensation. CNP analogs do not remove the causal FGFR3 gain-of-function; rather, they oppose an important downstream consequence of that signal. This distinction is relevant when interpreting outcomes beyond height velocity. A sustained increase in annualized growth velocity is clinically meaningful, but growth velocity alone does not capture the full clinical burden of achondroplasia. Longer-term vosoritide data suggest improvements in body proportionality in younger children, while the navepegritide trial reported exploratory improvements in lower-limb alignment and physical functioning [12,13]. However, evidence remains insufficient to determine whether current therapies reduce major complications such as foramen magnum stenosis, spinal stenosis, obstructive sleep apnea, or the need for orthopedic and neurosurgical procedures. Final adult height data are also not yet available for vosoritide. Thus, in the current clinical setting, “disease modification” primarily reflects the modification of growth-plate signaling and the growth phenotype and should not be interpreted as established prevention of the full spectrum of achondroplasia-related complications.

### 4.2. Small-Molecule Modulators: FGFR Kinase Inhibition and Repurposing

The direct inhibition of FGFR3 kinase activity targets the primary molecular lesion in achondroplasia. Infigratinib (BGJ398) is an orally bioavailable FGFR1-3 tyrosine-kinase inhibitor with low-nanomolar potency [16]. In achondroplasia-like mouse models, FGFR kinase inhibition with BGJ398 improved longitudinal bone growth and partially normalized growth-plate architecture, providing a rationale for clinical evaluation [17,18]. In a phase 2 study in children with achondroplasia (NCT04265651; completed 2025), oral infigratinib increased annualized height velocity and height z score without an apparent major safety signal in the reported follow-up, showing that FGFR kinase inhibition can increase growth in children with ACH [19]. Key translational questions include the therapeutic index during multi-year pediatric treatment, potential on-target effects in non-cartilage tissues, and whether greater FGFR3 selectivity can widen the safety margin.

Additional phase 3 evidence has since become available. In the randomized, double-blind, placebo-controlled PROPEL 3 trial, 114 children with achondroplasia received once-daily oral infigratinib or placebo for 52 weeks; treatment resulted in a significantly greater increase from baseline in annualized height velocity than placebo, with a least-squares mean difference of 1.74 cm/year (95% CI, 1.31 to 2.17; *p* < 0.001), and also improved height z score [20]. These findings strengthen the evidence that the pharmacological attenuation of FGFR signaling is clinically active in achondroplasia, although longer follow-up is needed to assess proportionality, skeletal complications, and safety during chronic pediatric exposure.

Second-generation, FGFR3-selective inhibitors aim to improve the therapeutic index by minimizing the inhibition of FGFR1 and FGFR2. Dabogratinib (formerly TYRA-300) [21] is an orally bioavailable FGFR3-selective inhibitor that increased bone growth in two FGFR3-driven mouse models of chondrodysplasia, including an achondroplasia-relevant model, and improved skeletal measures related to clinically important complications, providing a preclinical rationale for clinical evaluation [22]. Dabogratinib is currently being evaluated in BEACH301 (NCT06842355), an ongoing phase 2, open-label, dose-escalation and dose-expansion study in children aged 3–10 years with achondroplasia and open growth plates [23]. Clinical efficacy data in children with achondroplasia have not yet been published, and the study will determine whether greater FGFR3 selectivity translates into a favorable long-term safety and efficacy balance.

A parallel strategy is repurposing drugs that attenuate relevant signaling outputs in chondrocytes. Meclizine (also spelled meclozine), a first-generation H1 antihistamine used for motion sickness [24], was reported to attenuate elevated FGFR3 signaling in preclinical achondroplasia models [25]. Early clinical studies evaluated pharmacokinetics and safety in children with achondroplasia [26,27]. In a small open-label phase 2 study of nine children receiving meclizine together with growth hormone, height velocity increased only slightly, from 4.35 ± 1.36 to 4.46 ± 1.54 cm/year over 26 weeks, without evidence of an additive effect on linear growth [28]. Meclizine remains investigational, and the current clinical evidence is much more limited than for CNP-based therapies or FGFR kinase inhibitors.

Statins have also been explored as a repurposing strategy. Yamashita et al. showed that statin treatment rescued the abnormal cartilage phenotype in achondroplasia patient-derived iPSC models and improved bone growth in an achondroplasia mouse model. Lovastatin was used in the iPSC experiments, whereas rosuvastatin was administered to mice. These findings provided preclinical evidence for statins as potential FGFR3-modulating agents, but no clinical efficacy studies in achondroplasia have been reported [29].

These pharmacological approaches show the spectrum between target-proximal and target-indirect intervention. Infigratinib is mechanistically close to the disease driver because it inhibits FGFR kinase activity, whereas meclizine is a repurposed agent with a less direct relationship to FGFR3 signaling. FGFR3-selective compounds such as dabogratinib may represent the next step in this logic, because improved receptor selectivity could reduce the unwanted inhibition of other FGFR family members while preserving the desired effect in growth-plate chondrocytes. Combination approaches may also be considered because different components of FGFR3–NPR2 signaling can be targeted simultaneously. Direct evidence for combined CNP analog and FGFR kinase inhibition in achondroplasia is currently lacking. However, in a related preclinical study, the inhibition of FGF-induced NPR2 dephosphorylation with LB-100 enhanced the bone-growth effect of BMN-111, providing proof-of-principle for combination strategies [30]. Nevertheless, long-term pediatric use remains the central question for all small-molecule approaches. A growth-promoting signal must be maintained during a developmental period, but systemic exposure will also reach tissues in which FGFR signaling has normal physiological roles. Therefore, clinical translation depends not only on demonstrating increased growth velocity, but also on defining dose windows that are safe, durable, and compatible with many years of treatment [6,19].

## 5. Second-Wave Protein Biologics and Extracellular Intervention Points

### 5.1. FGFR3 Ligand Traps and Decoy Receptors

Recifercept (TA-46) is a soluble FGFR3 decoy receptor designed to sequester FGF ligands and reduce the activation of endogenous FGFR3. In preclinical studies, soluble FGFR3 constructs improved bone growth in achondroplasia mouse models [31]. However, in the pediatric phase 2 study, growth outcomes at 6 and 12 months were comparable with modeled untreated trajectories across all tested doses, indicating no measurable treatment effect and leading to the termination of clinical development [32,33]. The biological reason for this lack of efficacy has not been established.

### 5.2. FGF2 Targeting and Related Approaches

Fibroblast growth factor 2 (FGF2) is among the ligands capable of activating FGFR signaling in cartilage and has been implicated as a relevant driver of pathological FGFR3 pathway output in achondroplasia models [34]. RBM-007 (umedaptanib pegol) is a pegylated RNA aptamer that binds FGF2 and has also undergone early clinical evaluation in ophthalmology [35]. In a mouse model of FGFR3-related skeletal dysplasia/achondroplasia, the subcutaneous administration of RBM-007 restored defective skeletal growth and improved endochondral bone growth and growth-plate pathology [36]. An early phase 2 study of RBM-007 in children with achondroplasia has been completed in Japan, and a long-term phase 2 study is no longer recruiting participants [37,38]. A phase 3 study (jRCT2061260037) began enrollment in July 2026 [39]. RBM-007 has also been reviewed as a multi-indication anti-FGF2 platform [40], but peer-reviewed clinical efficacy data in achondroplasia have not yet been published; efficacy conclusions should therefore remain preliminary.

Vofatamab (B-701) is a fully human monoclonal antibody directed against the extracellular domain of FGFR3. It was clinically evaluated in oncology but did not advance to published preclinical or clinical studies in achondroplasia [34].

The experience with extracellular biologics is informative even where development has not led to an approved therapy. Ligand traps, aptamers, and receptor-blocking antibodies all test whether pathological FGFR3 output can be reduced without entering the cell or editing the genome. Their principal conceptual advantages are reversibility and the absence of permanent genomic modification. Their limitations include achieving adequate exposure in cartilage and the extent to which ligand neutralization or receptor blockade can reduce signaling from a gain-of-function receptor. For achondroplasia, these agents remain useful as investigational disease-modifying approaches and as mechanistic comparators for more durable genetic strategies [31,34,36].

The clinical development and efficacy evidence for the pharmacological and biological approaches discussed above are summarized in Table 1.

## 6. Toward Etiology-Directed and Genetic Therapies: Perspectives from Genome Editing

### 6.1. Why Genetic Strategies Are Attractive in Achondroplasia

Achondroplasia is caused by a single, well-defined gain-of-function variant, making it an appealing candidate for genetic intervention. However, the genetic approaches discussed below remain preclinical or conceptual and have not yet been evaluated clinically in patients with achondroplasia.

Three principal strategies can be considered: precise correction of the pathogenic allele, selective suppression of the mutant allele, or partial reduction of total FGFR3 expression in growth-plate cartilage. These approaches must be carefully titrated because excessive reduction of FGFR3 signaling may also disturb skeletal development. CATSHL syndrome (camptodactyly–tall stature–scoliosis–hearing loss) is associated with loss-of-function FGFR3 variants, although dominant forms involve specific missense variants and are not equivalent to simple FGFR3 haploinsufficiency [42]. Consistent with the physiological role of FGFR3 in restraining skeletal growth, Fgfr3-null mice exhibit an overgrowth of long bones and vertebrae together with hearing impairment [43]. These observations support controlled rather than maximal FGFR3 suppression, although they do not define a quantitative safe threshold for therapeutic down-regulation. Any clinically credible genetic strategy must therefore deliver quantitatively controlled modulation in cartilage, with a safety margin suitable for pediatric use.

This also defines the difference between gene therapy for achondroplasia and many classical gene-replacement indications. In loss-of-function diseases, adding a functional copy of a gene or supplying a missing protein can directly compensate for the defect. In achondroplasia, the problem is a gain-of-function receptor, so simple gene addition would not be therapeutic unless the added product antagonized the mutant pathway. Genetic strategies must therefore reduce, correct, or rebalance the activity of the existing FGFR3 signal. This makes allele specificity, quantitative control, and tissue restriction more important than high-level transgene expression alone [1,4,42].

### 6.2. Proof-of-Concept Genome Correction Studies and Their Limits

Proof-of-concept studies demonstrate that the achondroplasia allele can be corrected in cellular or early developmental contexts. Patient-derived induced pluripotent stem cells carrying FGFR3 p.Gly380Arg have been gene-edited to correct the mutation, restoring chondrogenic differentiation capacity in vitro and supporting the causal link between genotype and cellular phenotype [44]. In mouse experiments, the CRISPR-Cas9 correction of the corresponding Fgfr3 mutation has been demonstrated at the embryo/zygote stage with measurable correction and phenotypic improvement in edited offspring [45]. These embryo/zygote experiments represent developmental proof-of-concept rather than a clinically relevant therapeutic approach; the translational strategies considered below refer to postnatal somatic intervention.

Notably, achondroplasia is unusually attractive for precision editing because the vast majority of affected individuals carry the same p.Gly380Arg amino-acid substitution, arising predominantly from c.1138G>A and more rarely from c.1138G>C. This makes DSB-free CRISPR modalities conceptually appealing—adenine base editing [46] for the common c.1138G>A allele and prime editing for either allele, including c.1138G>C [47].

These studies mentioned above establish molecular feasibility, but they do not directly solve the central translational problem: safe and efficient somatic delivery to growth-plate chondrocytes in a developing child.

Somatic genome editing would require high on-target activity in a physically protected niche, the minimization of off-target edits and structural genomic alterations, and an effect durable enough to influence years of longitudinal growth but controllable enough to avoid long-term over-suppression. These constraints make quantitatively tunable gene-regulation strategies particularly attractive because they may be implemented without permanent genome breaks.

Although base and prime editing avoid programmed DSBs, their therapeutic use still requires a careful evaluation of editing efficiency, bystander changes, off-target activity, and the persistence of editor exposure in target and non-target tissues [46,47]. Successful editing may not need to occur in every chondrocyte because growth-plate function is a tissue-level process. However, the minimum fraction and spatial distribution of corrected or down-tuned cells needed for clinically meaningful benefit remain unknown. This uncertainty is one reason why expression-tuning strategies are attractive: they may allow a partial normalization of signaling tone without requiring the perfect repair of the pathogenic nucleotide in all target cells [48].

### 6.3. Targeting Regulatory DNA and Epigenetic Control of FGFR3 Expression

A promising alternative to single-base correction is to modulate FGFR3 expression by targeting cartilage-specific regulatory elements. Recently, the deletion of a cartilage-active enhancer located approximately 29 kb upstream of Fgfr3 (–29E) markedly improved skeletal features across the body in an achondroplasia mouse model [48]. In wild-type mice, homozygous –29E deletion reduced Fgfr3 expression to approximately 61% of control levels in long-bone epiphyses and 55% in the thoracic cage without an overt adverse skeletal phenotype. In the achondroplasia model, the deletion of –29E from the mutant allele or from both alleles substantially improved skeletal abnormalities, whereas deletion from the wild-type allele alone did not. These findings support the feasibility of partial FGFR3 down-regulation, although the optimal degree of suppression in human growth plates remains unknown. Importantly, the –29E deletion in this study was germline and therefore represents developmental proof-of-principle rather than a demonstration of postnatal somatic targeting. The corresponding human −29E region, located approximately 29 kb upstream of FGFR3, is highly conserved and shows epigenomic features consistent with enhancer activity in human fetal cartilage, but its regulatory function has not been directly validated in human cartilage [48].

Taken together, these findings provide preclinical proof-of-principle for “expression-tuning” approaches: rather than correcting the single nucleotide, one can reduce the effective FGFR3 signaling burden within cartilage to a more physiological range.

Regulatory-element targeting has three advantages. First, it can be allele-agnostic (useful even for rarer FGFR3 variants). Second, it naturally supports partial rather than absolute suppression, aligning with the need to avoid FGFR3 loss-of-function phenotypes [42]. Third, a cartilage-restricted enhancer can provide an intrinsic specificity layer, as illustrated by the −29E study in mice [48], although broad and predictable growth-plate exposure remains the key translational challenge.

The mouse data also suggest that partial, tissue-restricted reduction of Fgfr3 can improve both axial and appendicular skeletal phenotypes [48]. However, the magnitude and spatial distribution of repression required in human growth plates remain unknown.

The –29E result also fits within a broader regulatory-genomics framework. A 2024 enhancer map of pre-hypertrophic chondrocytes identified cartilage-active enhancers near genes involved in skeletal development and adult height variation; targeted deletions of selected enhancers at the Fgfr3, Col2a1, Hhip and Nkx3-2 loci confirmed their roles in regulating cognate genes [49]. Thus, candidate cis-regulatory targets for achondroplasia do not need to be selected only by single-gene intuition, but can be prioritized using enhancer maps and functional validation.

A permanent enhancer deletion is not the only way to use this principle. CRISPR interference (CRISPRi) could provide transcriptional repression without cutting DNA, whereas methylation-based epigenome editing could establish more durable silencing. Both strategies are conceptually compatible with partial FGFR3 down-tuning, but their durability, reversibility, and redosing requirements differ [50,51]. They would still require sufficient growth-plate delivery, tissue restriction, and quantitative control.

Technical precedents from other tissues support the plausibility of DSB-free transcriptional repression in vivo. AAV-compatible dSaCas9-KRAB systems have achieved systemic in vivo repression of Pcsk9 in adult mice, with reductions in serum Pcsk9/cholesterol and repression maintained for 24 weeks after one treatment [50]. More recently, an mRNA/LNP-delivered epigenetic editor targeting PCSK9 induced DNA-methylation-mediated silencing without changing the DNA sequence; in mice, silencing persisted for at least 1 year, and PCSK9 and LDL cholesterol were durably reduced after a single administration in cynomolgus monkeys [51]. In that study, reversal required a separate targeted demethylating activator. These studies are not cartilage studies, but they show that durable gene down-regulation can be achieved in vivo without nuclease-mediated DNA cutting. Unlike the largely non-dividing hepatocytes in these durability studies, growth-plate chondrocytes are actively proliferating and subsequently undergo hypertrophic differentiation. Therefore, the persistence of epigenetic repression through growth-plate cell turnover remains uncertain. Consequently, the need and timing of redosing cannot currently be predicted.

### 6.4. Delivery to Cartilage and Growth Plate: What Is Known and What Remains Unsolved

Somatic genetic therapy for achondroplasia is ultimately a delivery problem. Cartilage tissue is avascular and dominated by a dense, highly anionic extracellular matrix that limits convective transport and imposes strong size-/charge-dependent diffusion barriers [52]. In vivo imaging of the murine growth plate further demonstrated a strong size-dependent delivery barrier: 3 kDa dextran reached approximately 60% and 10 kDa dextran approximately 10% of vascular fluorescence within the growth plate, whereas 40 kDa and larger dextrans showed no detectable entry within a detection limit of a few percent of vascular concentration [53]. A central design principle emerging from articular-cartilage drug delivery is to exploit electrostatic interactions with sulfated glycosaminoglycans to enhance penetration and to retain payloads within the matrix, for example, using cationic carriers that achieve rapid uptake and sustained intra-cartilage residence after intra-articular dosing [54]. In parallel, multiple targeting chemistries (including cartilage-binding peptides and nanoparticle surface modifications) have been developed to bias localization to cartilage and improve residence time in joint models [55,56].

For nucleic-acid therapeutics, there is growing evidence that appropriately engineered nonviral systems can access chondrocytes and support functional expression within cartilage. One example is intra-articular lipid nanoparticles delivering mRNA with measurable expression and deeper cartilage penetration than recombinant proteins in vivo [57]. Engineered exosome–liposome hybrid systems also delivered CRISPR cargo to chondrocytes in rodent osteoarthritis models, demonstrating cartilage-cell targeting and on-tissue gene modulation [58]. Although articular cartilage differs from growth-plate cartilage in structure, remodeling dynamics, and access, these studies provide concrete proof-of-principle that vesicle-guided targeting, cargo protection, and local retention can enable functional delivery to cartilage cell populations. More directly relevant to growth-plate delivery, cartilage-targeted exosomes carrying siRNA and growth hormone showed targeted delivery to growth-plate cartilage after systemic administration in immature rats [59]. In an FGFR3-related hypochondroplasia mouse model, intravenously administered WYRGRL-modified, chondrocyte-membrane-coated nanoparticles accumulated in distal femoral growth-plate regions, with peak accumulation at approximately 24 h and detectable signal through 72 h [60]. These studies provide proof-of-principle for systemic growth-plate targeting, but neither establishes the broad and quantitatively uniform coverage of multiple growth plates throughout the skeleton.

Despite these advances, much of the delivery evidence base remains derived from intra-articular studies of articular cartilage, while the available systemic studies do not yet demonstrate broad, predictable access to multiple growth plates throughout the skeleton. Viral delivery has been explored extensively in joint settings as well; several studies have compared AAV serotypes for chondrocyte/cartilage transduction and have pursued capsid engineering to improve joint tropism and reduce off-target biodistribution after intra-articular dosing [61,62,63]. Nevertheless, systemic delivery to growth plates remains a distinct translational barrier. Bone-targeting studies demonstrate that skeletal delivery is feasible [64], but do not by themselves establish the efficient transduction of growth-plate chondrocytes.

Taken together, the joint-cartilage literature shows that local access to cartilage cells is feasible, but achondroplasia requires the broad and predictable exposure of multiple growth plates, including clinically important cranial-base and vertebral regions [1,4]. Biodistribution studies should therefore distinguish vascularized bone, marrow, articular cartilage, and growth-plate cartilage rather than report bulk “bone” measurements. The relevant cellular targets are proliferative and prehypertrophic chondrocytes, not the skeleton as a whole [6,48]. Achieving this goal will require dedicated platform engineering for tropism, dose control, and cargo format. Reversible gene-regulation formats may add a safety layer compared with permanent editing, but they also increase the need for repeat dosing.

The choice of cargo is inseparable from the delivery platform, and each system involves different trade-offs. AAV vectors can support relatively durable expression, but their limited cargo capacity and anti-capsid immunity complicate the delivery of large editing systems and potential redosing [65]. LNPs enable transient RNA delivery, but the available cartilage-directed evidence remains largely based on local administration [57]. Engineered exosomes can carry nucleic-acid cargo and incorporate cartilage-targeting ligands, with systemic growth-plate targeting demonstrated in animal models [59], although broad skeletal coverage remains unproven. ASO and siRNA approaches are reversible and dose-adjustable but generally require repeated administration, while efficient delivery to target tissues remains a major limitation [66]. RNP delivery can similarly limit prolonged editor exposure, but does not remove the need for efficient intracellular delivery to growth-plate chondrocytes. Across these platforms, no approach has yet demonstrated broad, predictable systemic delivery to the multiple growth plates required for the treatment of achondroplasia.

The main genetic and gene-regulatory strategies considered for achondroplasia are compared in Table 2.

### 6.5. Safety, Ethics, and Translational Bottlenecks

Etiology-directed genetic therapies in children face particularly high safety thresholds. Key risks include off-target genome modification, chromosomal rearrangements from double-strand breaks, immune responses to delivery vectors or gene-editing proteins, and unintended effects on other FGFR3-expressing tissues. An uneven or mosaic modification of different growth plates could also result in unequal skeletal responses or asymmetric growth. Unintended genomic alterations may also create long-term genotoxicity and tumorigenicity risks, requiring extended follow-up [67]. Because achondroplasia is compatible with long life expectancy, clinical translation will require extensive preclinical safety and biodistribution studies, standardized manufacturing and quality control, and long-term follow-up appropriate for pediatric gene therapy. Germline editing is neither ethically acceptable nor clinically relevant for ACH treatment. By contrast, preimplantation genetic testing for monogenic disorders (PGT-M) is an established reproductive option for families in whom an FGFR3 pathogenic variant has been identified [4].

For these reasons, the most credible near-term “genetic” path may be the controlled down-tuning of FGFR3 expression or signaling in cartilage using tissue-guided delivery and reversible platforms, rather than a permanent correction of the pathogenic nucleotide [48,50,51]. In addition, genetic strategies should be evaluated not only for height velocity but also for effects on complications driven by disproportionate skeletal anatomy (e.g., foramen magnum stenosis, spinal stenosis, and limb alignment), which may require earlier and more comprehensive growth-plate engagement than pharmacological approaches aimed primarily at linear growth.

A useful development path may therefore start with reversible or dose-adjustable modalities before moving toward permanent editing. In early studies, endpoints should include molecular pathway readouts, growth velocity, proportionality, and imaging-based markers of clinically relevant complications. Because many complications of achondroplasia arise from disproportionate skeletal development rather than only from short stature, a therapy that increases height without improving craniofacial, spinal, or limb-alignment outcomes may have limited disease-modifying value. The goals of treatment are not viewed uniformly within the dwarfism community, and treatment priorities may differ among affected individuals and families [68]. Little People of America has emphasized that increased growth velocity alone should not be equated with improved health or quality of life, underscoring the importance of distinguishing growth-promoting effects from the prevention of clinically important complications [69]. Because disease-modifying treatment may begin in young children and continue for several years, treatment decisions should involve parental permission and, when developmentally appropriate, the child’s assent, within a shared decision-making process that takes individual and family priorities into account. Conversely, a modest effect on height could still be clinically important if it reduces high-risk anatomical complications. This argues for trial designs and preclinical models that evaluate skeletal architecture broadly, not only linear growth [1,8,12].

## 7. Conclusions

The therapeutic landscape for achondroplasia has moved beyond supportive care and now includes mechanism-based disease-modifying options. NPR2/CNP pathway activation is established in clinical practice with daily vosoritide. Navepegritide has shown efficacy in a controlled trial and received FDA accelerated approval, providing a once-weekly alternative. Oral FGFR inhibition with infigratinib has increased annualized height velocity in both phase 2 and phase 3 studies, while the FGFR3-selective inhibitor dabogratinib has entered phase 2 clinical evaluation.

At the same time, it is still uncertain how much these gains in linear growth will translate into patient-relevant outcomes. Some trials include exploratory signals for lower-limb alignment and patient-reported physical functioning, but effects on major complications (foramen magnum stenosis and related neurosurgical interventions, spinal stenosis, obstructive sleep apnea, and orthopedic surgery rates) will require dedicated endpoints and longer-term follow-up.

From the perspective of genome-editing research, achondroplasia remains an attractive target for etiology-directed genetic therapy because of its well-defined molecular cause, but delivery to growth plates remains the key limitation. Cartilage-biased gene regulation approaches, including enhancer targeting and CRISPRi-like modulation, represent promising experimental directions, but remain substantially less mature than clinically advanced CNP-axis therapies and pharmacological FGFR inhibition. Progress will likely depend on early-life intervention, careful quantitative titration to avoid over-suppression, and outcome measures that capture both growth and clinically meaningful complications.

## Figures and Tables

**Figure 1:**
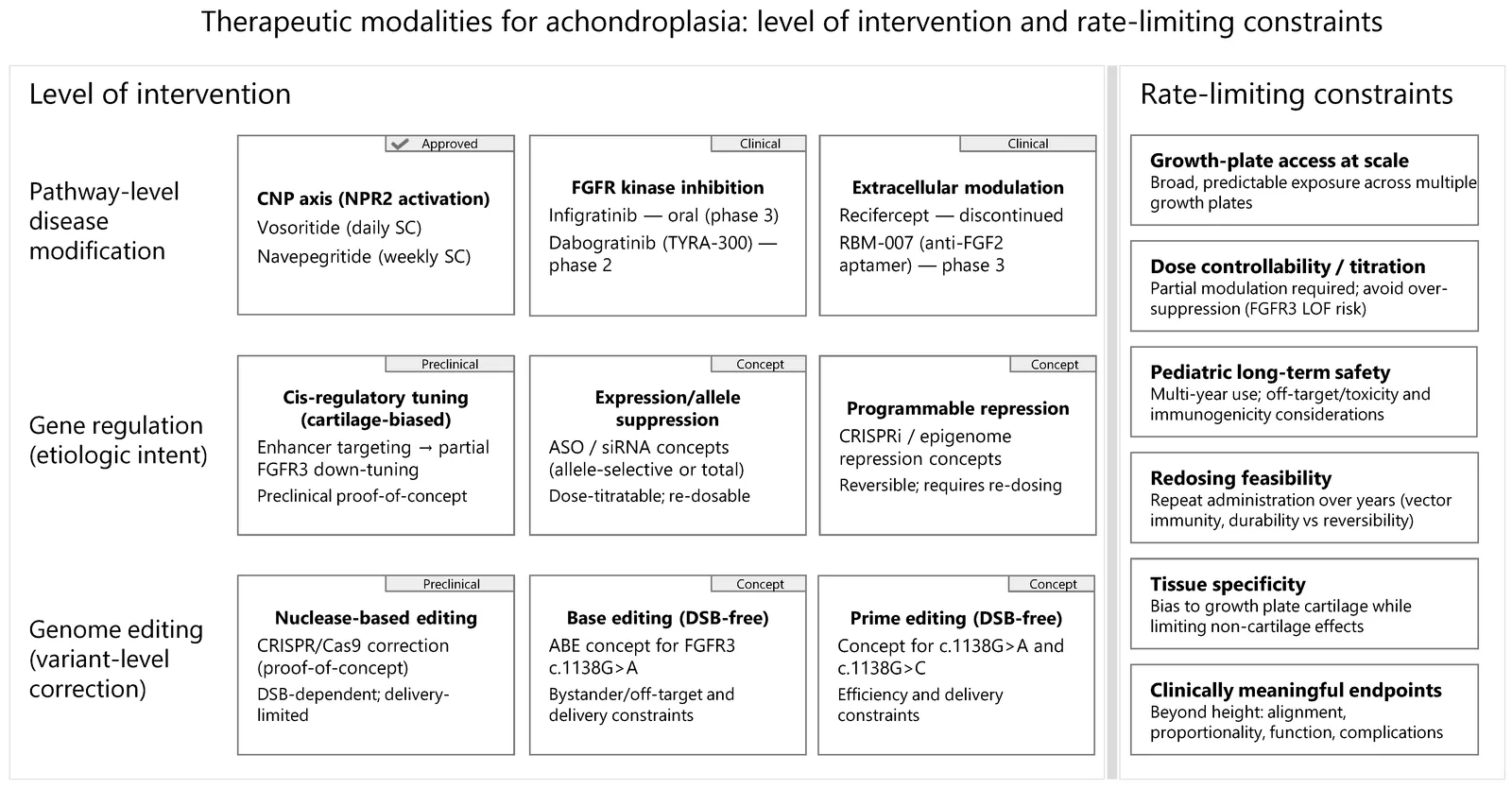
Therapeutic modalities for achondroplasia mapped to level of intervention and rate-limiting constraints. The left panel organizes current and emerging approaches by level of intervention, from pathway-level disease modification with clinical evidence (NPR2/CNP pathway activation, FGFR kinase inhibition, and extracellular modulation) to gene regulation strategies with etiologic intent and variant-level genome editing concepts. Evidence maturity is indicated by the status labels (approved, clinical, early clinical, preclinical, concept). The right panel lists the main rate-limiting constraints relevant across modalities. Abbreviations: ABE, adenine base editor; ASO, antisense oligonucleotide; CNP, C-type natriuretic peptide; CRISPRi, CRISPR interference; DSB, double-strand break; FGF2, fibroblast growth factor 2; FGFR3, fibroblast growth factor receptor 3; LOF, loss of function; NPR2, natriuretic peptide receptor 2; SC, subcutaneous; siRNA, small interfering RNA.

**Table 1:** Clinical development and efficacy evidence for pharmacological and biological therapies in achondroplasia.

Agent	Mechanism/Level of Action	Route/Dosing	Highest Stage/Key Trial	Principal Clinical Efficacy Result	Other Clinically Relevant Findings	Current Status	Key Refs.
Vosoritide (BMN 111; Voxzogo)	CNP analog; NPR2 activation and functional antagonism of FGFR3–MAPK signaling	SC, once daily (15 μg/kg)	Approved; phase 3, NCT03197766	Adjusted mean difference in annualized growth velocity vs. placebo: +1.57 cm/year at 52 weeks (95% CI, 1.22–1.93; *p* < 0.0001)	Sustained growth-promoting effect reported during long-term follow-up; effects on final adult height and major ACH complications remain uncertain	Approved disease-modifying therapy	[8,9,10,11,12]
Navepegritide (TransCon CNP; Yuviwel)	Long-acting CNP prodrug; NPR2 activation	SC, once weekly	FDA accelerated approval; phase 2b APPROACH, NCT05598320	LS mean difference in annualized growth velocity vs. placebo: +1.49 cm/year at 52 weeks (95% CI, 1.05–1.93; *p* < 0.001)	Improvements were reported in tibiofemoral angle, mechanical axis deviation, fibula-to-tibia length ratio, and an exploratory physical-function measure	FDA accelerated approval (2026); confirmatory clinical benefit remains to be established	[13,14]
BMN 333	Long-acting CNP analog; NPR2 activation	SC; investigational long-acting regimen	Phase 2/3 ASPEN, NCT07441876	No clinical efficacy data in children with ACH published to date	Phase 3 is designed to compare BMN 333 directly with vosoritide	Ongoing phase 2/3; recruiting	[15]
Infigratinib (BGJ398)	FGFR1–3 tyrosine-kinase inhibitor; direct attenuation of FGFR signaling	Oral, once daily (0.25 mg/kg in phase 3)	Phase 3 completed; PROPEL 3, NCT06164951	LS mean difference in change from baseline in annualized height velocity vs. placebo: +1.74 cm/year at 52 weeks (95% CI, 1.31–2.17; *p* < 0.001)	Height z score improved; the between-group difference in upper-to-lower body segment ratio was −0.02 (96% CI, −0.06 to 0.01)	Investigational; positive phase 3 results, not FDA-approved for ACH as of August 2026	[19,20]
Dabogratinib (TYRA-300)	FGFR3-selective tyrosine-kinase inhibitor	Oral, once daily; dose escalation	Phase 2 BEACH301, NCT06842355	No clinical efficacy data in ACH published to date	No clinical non-growth outcomes available; preclinical studies showed improvement in skeletal measures	Ongoing phase 2	[22,23]
Meclizine (meclozine)	Repurposed H1 antihistamine; indirect attenuation of FGFR3-related signaling	Oral, once daily; 12.5 or 25 mg/day in phase 2, concomitantly with growth hormone	Phase 2, jRCT2041230001	Height velocity changed from 4.35 ± 1.36 to 4.46 ± 1.54 cm/year after 26 weeks; no additive effect on linear growth was demonstrated	Arm-span velocity was 6.93 ± 2.50 cm/year; this finding was exploratory	Investigational; clinical evidence remains limited	[26,27,28]
Recifercept (TA-46)	Soluble FGFR3 decoy receptor; extracellular ligand sequestration	SC; weekly, twice-weekly and daily regimens evaluated	Phase 2, NCT04638153; terminated	Prespecified efficacy criteria were not met; the study was terminated because of lack of efficacy at the tested doses	Termination was not related to a safety concern	Development discontinued for ACH	[31,32,33,41]
RBM-007 (umedaptanib pegol)	PEGylated RNA aptamer targeting FGF2; extracellular reduction of ligand-driven FGFR signaling	SC; weekly or biweekly regimens evaluated in phase 2	Phase 2 completed, jRCT2031220291; phase 3, jRCT2061260037	No peer-reviewed clinical efficacy data published to date.	No peer-reviewed data on non-growth clinical outcomes published to date.	Phase 3 ongoing (initiated July 2026)	[36,37,38,39,40]
Vofatamab (B-701)	Fully human anti-FGFR3 monoclonal antibody; extracellular receptor blockade	Not evaluated in ACH	No published ACH clinical studies	No clinical efficacy data in ACH	No published preclinical ACH studies were identified in the cited review	No ACH-specific clinical development reported	[34]
Statins (e.g., lovastatin, rosuvastatin)	Repurposed HMG-CoA reductase inhibitors; indirect modulation of FGFR3-related chondrocyte dysfunction	Oral clinically as a drug class; experimental dosing in ACH models	Preclinical	No clinical efficacy data in ACH	Rescue of cartilage phenotype in ACH patient-derived iPSCs; rosuvastatin improved bone growth in an ACH mouse model	Preclinical; not clinically developed for ACH	[29]

**Table 2:** Potential genetic and gene-regulatory strategies for achondroplasia and their translational limitations.

Strategy	Potential Use in ACH	DSB Reliance	Reversibility	Redosing Capability	Evidence in ACH	Key Modality-Specific Limitation
Nuclease editing	Correction/disruption of mutant *FGFR3* allele	Yes	No	Generally not intended	ACH iPSC and embryo/zygote proof-of-concept	DSB-associated genomic alterations and irreversible editing
Base editing	Correction of the common c.1138G>A allele	No programmed DSB	No	Generally not intended	Conceptual for ACH; no somatic ACH study	Bystander/off-target editing and restricted editable sequence context
Prime editing	Correction of c.1138G>A or c.1138G>C	No programmed DSB	No	Generally not intended	Conceptual for ACH	Lower/variable editing efficiency and complex cargo requirements
CRISPRi/epigenome editing	Partial down-regulation of *FGFR3* or cartilage-active regulatory elements	No programmed DSB	Potentially reversible/variable durability	Potentially	No direct ACH therapeutic study; supported by enhancer biology	Uncertain durability and quantitative control in proliferating growth-plate chondrocytes
ASO/siRNA	Transient reduction of *FGFR3* transcript	No	Yes	Yes	No established ACH efficacy	Transient effect and need for repeated administration

## Data Availability

No new data were created or analyzed in this study. Data sharing is not applicable to this article.
